# Overview of DrugProt task at BioCreative VII: data and methods for large-scale text mining and knowledge graph generation of heterogenous chemical–protein relations

**DOI:** 10.1093/database/baad080

**Published:** 2023-11-28

**Authors:** Antonio Miranda-Escalada, Farrokh Mehryary, Jouni Luoma, Darryl Estrada-Zavala, Luis Gasco, Sampo Pyysalo, Alfonso Valencia, Martin Krallinger

**Affiliations:** Life Sciences Department, Barcelona Supercomputing Center, Barcelona 08034, Spain; TurkuNLP Group, Department of Computing, University of Turku, Turku 20014, Finland; TurkuNLP Group, Department of Computing, University of Turku, Turku 20014, Finland; Life Sciences Department, Barcelona Supercomputing Center, Barcelona 08034, Spain; Life Sciences Department, Barcelona Supercomputing Center, Barcelona 08034, Spain; TurkuNLP Group, Department of Computing, University of Turku, Turku 20014, Finland; Life Sciences Department, Barcelona Supercomputing Center, Barcelona 08034, Spain; Life Sciences Department, Barcelona Supercomputing Center, Barcelona 08034, Spain

## Abstract

It is getting increasingly challenging to efficiently exploit drug-related information described in the growing amount of scientific literature. Indeed, for drug–gene/protein interactions, the challenge is even bigger, considering the scattered information sources and types of interactions. However, their systematic, large-scale exploitation is key for developing tools, impacting knowledge fields as diverse as drug design or metabolic pathway research. Previous efforts in the extraction of drug–gene/protein interactions from the literature did not address these scalability and granularity issues. To tackle them, we have organized the DrugProt track at BioCreative VII. In the context of the track, we have released the DrugProt Gold Standard corpus, a collection of 5000 PubMed abstracts, manually annotated with granular drug–gene/protein interactions. We have proposed a novel large-scale track to evaluate the capacity of natural language processing systems to scale to the range of millions of documents, and generate with their predictions a silver standard knowledge graph of 53 993 602 nodes and 19 367 406 edges. Its use exceeds the shared task and points toward pharmacological and biological applications such as drug discovery or continuous database curation. Finally, we have created a persistent evaluation scenario on CodaLab to continuously evaluate new relation extraction systems that may arise. Thirty teams from four continents, which involved 110 people, sent 107 submission runs for the Main DrugProt track, and nine teams submitted 21 runs for the Large Scale DrugProt track. Most participants implemented deep learning approaches based on pretrained transformer-like language models (LMs) such as BERT or BioBERT, reaching precision and recall values as high as 0.9167 and 0.9542 for some relation types. Finally, some initial explorations of the applicability of the knowledge graph have shown its potential to explore the chemical–protein relations described in the literature, or chemical compound–enzyme interactions.

**Database URL:**  https://doi.org/10.5281/zenodo.4955410

## Introduction

The volume of drug-related information stored in scientific literature is growing continuously and it is challenging to exploit it efficiently. In particular, there is a range of different types of drug–gene/protein interactions, and their systematic extraction and characterization are essential to analyze, predict and explore key biomedical properties underlying high-impact biomedical applications. Indeed, protein–chemical interactions are key in cellular processes, and their study is central for applications such as drug discovery, adverse drug reactions, drug repurposing and drug design studies. Nevertheless, the existing information on protein–chemical interactions is dispersed across a large diversity of databases and literature repositories such as DrugProt (1), STITCH (2) and ChEMBL (3). Maintaining and updating this information within these databases poses a complex challenge for their administrators. Therefore, there is a pressing need to centralize and structure the fragmented literature data into annotated databases that specifically serve the domains of biology, pharmacology and clinical research, with the inclusion of natural language processing (NLP) methods to unlock the information embedded within the documents.

Relation extraction (RE) is an NLP task that concerns identifying and classifying relations/interactions between named entities extracted from texts. It comes after named entity recognition (NER) in information extraction pipelines. For instance, for the task of detecting protein–chemical interactions, a system must (i) recognize the protein and chemical mentions (NER) and (ii) identify and classify the described protein–chemical relation (RE).

ChemProt (4) has been the most popular open effort for extracting chemical–protein interactions from biomedical literature. Nevertheless, employing the outputs of ChemProt for practical applications reveals some limitations.

First, ChemProt combined several interaction relations into 10 categories (only five used for benchmarking). This issue posed a challenge from a granularity perspective, as those groups hindered the practical utility of the resource in biological applications. Furthermore, the grouping of the relations not only introduced complexity into the classification procedure but also created problems generating a consistent knowledge graph. Moreover, the challenge of generating a massive knowledge graph was also problematic because of the absence of scalability assessment within the ChemProt shared task.

The ChemProt results considerably impacted the development and evaluation of new biomedical RE systems. However, it reflected one of the barriers to the NLP development in clinical applications, as identified by Chapman *et al.* 2011: ‘Lack of user-centered development and scalability’ (5). Currently, biomedical information is scattered over the literature. Plus, systems must be evaluated in scenarios aligned with real-world applications and must scale up efficiently to large amounts of data from different sources and dates.

To address these three issues, we have organized the novel DrugProt shared task, which focuses on user-centered development and scalability. First, relation types are more granular and aligned with real-world applications. Second, we have selected high-impact relations associated with biological interaction networks for applications such as drug discovery. Third, a single entity pair may be associated with multiple relation types, as in biomedical literature. Lastly, we introduce the Large Scale DrugProt track that serves to evaluate the scalability of systems in terms of their predictive performance.

The output of the DrugProt shared task includes the largest, manually annotated corpus for chemical–protein interaction extraction with text-bound annotation mentions. With the mention annotations, we have trained the DrugProt NER taggers: two state-of-the-art NER systems (6), one for chemical and the other for gene/protein mentions. The model engine is public at GitHub (https://github.com/jouniluoma/drugprot-ner-tagger).

The NER systems were run on the entire PubMed. For the Large Scale DrugProt track, we have released a subset of it (2.3 million abstracts), the silver standard corpus of gene/protein mentions with 33 578 479 named entities and the silver standard corpus of drug mentions with 20 415 123 named entities. In addition, the participants’ predictions of the Large Scale DrugProt track have allowed the creation of a silver standard knowledge graph of gene/protein–drug relations over those 2.3M PubMed abstracts. Additionally, we have run a competitive RE system (Turku-BSC system) over the entire PubMed dataset, resulting in the creation of a massive knowledge graph of relations extracted from the whole PubMed (https://doi.org/10.5281/zenodo.7252237). This knowledge graph is highly relevant for a wide spectrum of applications that involve mining chemical–gene information, such as drug discovery, drug design, adverse drug reactions, drug repurposing studies or database curation.

Knowledge graphs are relevant, and recently there has been a significant increase in their generation within the context of the COVID pandemic. Examples include the work of Domingo-Fernández *et al.* (7), Wang *et al.* (8) or Shengtian *et al.* (9). However, it is noteworthy that the methods utilized for their creation were not at the cutting edge, mainly relying on lexical approaches or dictionary-based methods.

The generation of knowledge graphs within the DrugProt initiative involved the use of a state-of-the-art NER system (6) and a combination of leading-edge biomedical RE systems. Additionally, the DrugProt setting allows the granular benchmarking of such systems. Moreover, the NER system, akin to numerous RE systems, is accessible to the public.

### Methodological evolution in RE

Throughout the course of the DrugProt initiative, notable advancements have been observed in the performance of biomedical RE systems. To contextualize this progress, the following paragraphs offer a brief overview of the evolution of RE methods.

Given that RE follows NER in information extraction pipelines, entity mentions always serve as input data to create RE systems. This granularity in annotations enables task organizers to evaluate and compare the performance of the RE task independently, rather than evaluating the combined performance of the entity and RE tasks. Therefore, the typical RE scenario starts with a set of documents with annotated named entities (referred to as ‘mentions’), with the primary objective of identifying and classifying relations between those named entities. Developers can either utilize supervised machine learning (ML) techniques or, conversely, employ unsupervised methods.

Systems built using unsupervised techniques are constructed exclusively using the texts and named entities. These systems can leverage several techniques including pattern clustering (10), dependency parsing (11) or heuristics (12). However, these techniques usually require large-scale corpora as support, exhibit limitations in distinguishing between various relation types and often have a low recall when generating low-frequency relation pairs (13).

**Figure 1.**
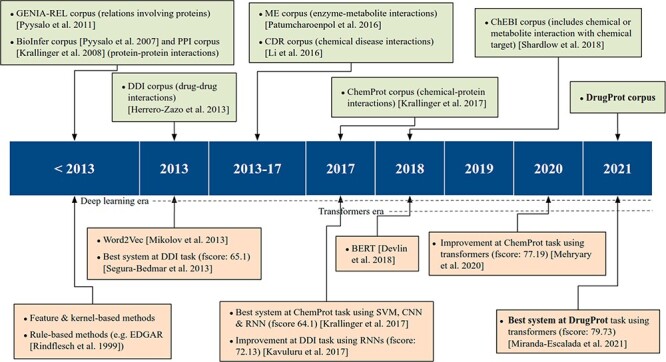
An overview of relevant RE corpora and technologies.

On the other hand, supervised systems need prelabeled example data to learn from. These training data facilitate the inference of model parameters and their use over previously unseen datasets. In this methodology, training data are of vital importance to achieve high-quality models. Consequently, the process of generating data with rigorous quality standards, following specific guidelines, becomes an essential step for both assessing and refining RE models. These corpora, which have been manually labeled by experts, are commonly known as Gold Standard (GS). However, due to manual annotation being laborious and expensive, an alternative is to generate automatic annotations with several automated systems and combine them. This corpus is typically called silver standard, a concept introduced by the CALBC initiative (14). The upper part of Figure 1 shows the most significant RE corpora over time.

Within the biomedical domain, there are several GS corpora with relation annotations. Attending to the entities involved in the relations, numerous open corpora focus on distinct categories. For instance, there are resources on protein–protein interactions, including the PPI (15) corpus used in BioCreative II and the BioInfer (16) corpus. Specialized corpora on drug–drug interactions, exemplified by the DDI corpus (17) released as part of SemEval2013; chemical diseased interactions, like the CDR corpus (18), a component part of the BioCreative V venue; or enzyme–metabolite interactions, such as ME corpus (19)), among many others.

ChemProt (4) is the most popular open GS for chemical–protein interactions. It contains PubMed abstracts exhaustively and manually annotated with mentions of chemical compounds/drugs and genes/proteins, as well as 22 different types of compound–protein relations. The corpus was published with the relations grouped into 10 classes, out of which 5 were utilized during the task evaluation process. It was employed in BioCreative VI and since then has been used as a standard benchmark for evaluating biomedical RE systems (20, 21).

In addition to ChemProt, other corpora include chemical–protein interactions among different relation types. For instance, the GENIA-REL corpus (22) focuses on relations involving proteins, and the ChEBI corpus (23) includes one relation-type tailoring when ‘chemical or metabolite interacts with and affects the behavior of a biological target’.

There are also chemical–protein interaction corpora created solely to evaluate a specific RE system. For instance, Humphreys *et al.* (24) created a corpus of seven articles from the journals *Biochimica et Biophysica Acta* and *FEMS Microbiology Letters*, and Czarnecki *et al.* (25) created a small training corpus of metabolic reaction information.

As previously discussed, GS corpora play an important role in advancing the state-of-the-art in RE, complementing technological progress. The release of these corpora has facilitated the creation of numerous supervised RE systems, as shown in the lower part of Figure 1. According to Bach *et al.* (26), up to 2013, ‘supervised approaches for RE were further divided into feature-based methods and kernel methods’.

In feature-based methods, syntactic and semantic features are extracted from the texts. Then, these features are input into an RE system to train it or to extract novel relations. Transforming the original text into the right features requires a lot of work and is one of the major bottlenecks of this approach. Kernel-based methods do not need the explicit definition of *a priori* features. Kernel functions use the original instance representation and compute similarities between a pair of instances (27). Therefore, the feature engineering workload is reduced, and the feature space can become much larger than the feature-based methods.

Kernel and feature-based methods were compared for biomedical RE in the SemEval DDI-2013 (28) shared task. At the time of the task, both feature and kernel-based approaches were used by competitive teams. Indeed, the highest performance was obtained by Chowdhury *et al.* (29) who designed a two-stage system. First, a feature-based classifier discards sentences with no relation. Second, a kernel-based system classifies the remaining sentences into one of the four relation types defined in the task. Regarding the ML algorithm choice, all participants employed support vector machines, and non-linear kernels were more successful than linear ones.

From 2013 on, artificial neural networks, which work based on dense vector representations, produced superior results on various NLP tasks, including RE. This was mainly due to the success of word embeddings (dense vector representation of words) and deep learning methods (30). Two major deep learning architectures have been initially employed in NLP tasks: recurrent neural networks (RNNs) and convolutional neural networks (CNNs). The input text is first tokenized and then encoded into a dense vector representation, using word embeddings, RNN and/or CNN layers. Then, the results can be fed to one or more non-linear transformation layers, which are finally followed by one or more classification layers.

In the DDI-2013 benchmark, while the best-performing 2013 system had reached a micro F1 of 65.1%, later approaches using different flavors of RNNs obtained micro *F*1-scores as high as 72.13 (31) and 72.55 (32). At BioCreative VI, the ChemProt shared task winners achieved an *F*-score of 64.1, by making an ensemble system composed of RNNs and CNNs with other ML architectures (33).

Recently, transformer-based architectures have become extremely popular, producing state-of-the-art results for various NLP tasks, including RE. It is very common to use large transformer-based pretrained LMs in the encoding step of the NLP systems, followed by simple decoding or classification layers. Some of the most common transformer-based pretrained LMs are BERT (34), BioBERT (20), SciBERT (35) and PubMedBERT (36). In the most usual paradigm, such pretrained transformers are fine-tuned, i.e. their weights are modified during training with the actual training data given for a particular task at hand. For example, by fine-tuning a pretrained BERT encoder on ChemProt training data, Lee *et al.* (20) achieved an *F*-score of 76.46 for this task. Similarly, Mehryary *et al.* (37) outperformed the previous results when they achieved an *F*-score of 77.19 by combining a BERT encoder with entity-pair embeddings.

## Materials and methods

### DrugProt corpus generation

We have released a large manually labeled corpus with (i) mentions of chemical compounds and drugs (named as CEMs throughout this paper), (ii) mentions of genes, proteins and miRNAs (named as GPROs throughout this paper) and (iii) relations between CEMs and GPROs.

**Figure 2.**
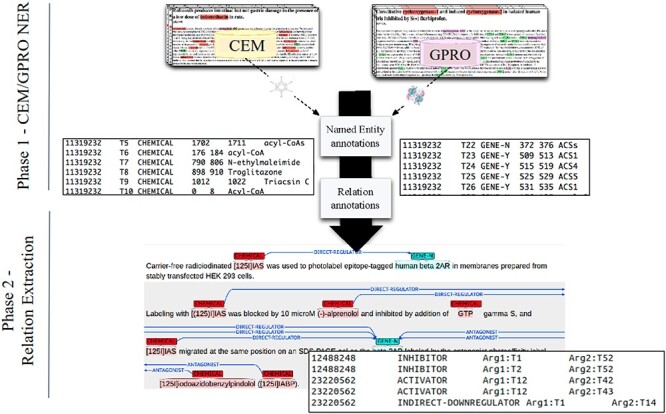
A DrugProt corpus annotation scheme in three independent phases: (1.1) CEM annotation, (1.2) GPRO annotation and (2) RE annotation. An example of the output of the annotation is visualized in Brat at the bottom of the figure.

These three annotation layers were performed independently on the same documents. First, CEMs were manually annotated to create the DrugProt chemical mention GS. Then, GPROs were manually annotated to create the DrugProt gene mention GS. Finally, both mention GSs were joined, and a third team of annotators marked the binary CEM–GPRO relations to create the DrugProt relation GS. (Figure 2). While marking the binary relations, annotators corrected a small percentage of wrongly annotated CEM and GPRO mentions.

The DrugProt corpus’s main goal is the training and evaluation of biomedical RE systems for extracting CEM–GPRO relations. Besides, it also serves to train biomedical NER systems of CEMs and GPROs. Indeed, the CEM and GPRO mention GSs are larger than most biomedical NER GSs.

The annotated texts consist of PubMed titles and abstracts in English from scientific papers published between 2005 and 2014. A subset of these abstracts comes from the previous ChemProt–BioCreative VI task, which included abstracts from the CHEMDNER–BioCreative IV task for the annotation of CEMs enriched with abstracts cited in the DrugBank database (38, 39). All the abstracts used in the previous ChemProt task were included in the training and development sets. In total, 5000 PubMed abstracts were manually annotated. Further statistics and details are provided in the Results section. The DrugProt corpus is available on Zenodo (https://doi.org/10.5281/zenodo.4955410).

#### DrugProt chemical mention GS

This GS contains the CEM mentions (chemical or drug entities), manually annotated following the DrugProt chemicals and drugs annotation guidelines. These guidelines were also employed in the CHEMDNER–Biocreative IV task (40). They were created after revising previous works, including but not limited to, the gene mention tasks of previous BioCreative efforts (41) and the annotation rules used by Kolaric *et al.* (42) and Corbett *et al.* (43). They were then refined through iterative cycles of annotations of sample documents. During this iterative process, annotators incorporated their suggestions and guideline inconsistencies were detected and solved by comparing the annotation differences of several annotators.

The annotation was carried out following the rules defined in the published annotation guidelines (https://doi.org/10.5281/zenodo.4957518). These guidelines define the criteria for identifying CEMs, which are those nouns of specific chemicals, specific classes of chemicals or fragments of specific chemicals. General chemical concepts, proteins, lipids and macromolecular biochemicals were excluded from the annotation scope. Finally, all mentions could be associated with chemical structure information to at least a certain degree of reliability. This implied that very general chemical concepts (non-structural or non-specific chemical nouns), adjectives, verbs and other terms (reactions and enzymes) were excluded from the annotation process.

Annotators were mainly organic chemistry postgraduates with an average experience of 3–4 years in the annotation of chemical names and chemical structures (44). This is necessary since the process requires extensive domain knowledge of chemistry, chemoinformatics or biochemistry to make sure the annotations are correct. The annotation was exclusively manual to prevent potential annotation biases that could arise from pre-annotation automated methods. The AnnotateIt tool (45) was employed as the application for carrying out this manual process.

To evaluate the quality of the corpus and the guidelines, different annotators labeled the same subset of documents following the same guidelines. Their parallel annotations were then compared to compute the Inter-Annotator Agreement (IAA). This score allows us to interpret how independent annotators apply the same guidelines and is a measure of task reproducibility and corpus quality. In the DrugProt chemical mention GS, an IAA measure was conducted on a subset of 100 documents, yielding a metric of 91% when assessing the exact match between mentions.

#### DrugProt gene mention GS

This corpus contains the PubMed abstracts manually annotated with GPROs [mentions of genes, gene products (proteins and RNAs), DNA/protein sequence elements and protein families, domains and complexes]. The annotation was carried out following the DrugProt gene and protein annotation guidelines (https://doi.org/10.5281/zenodo.4957576), which were previously employed in the CHEMDNER-patents track of BioCreative V.II. For the preparation of the guidelines, many previous corpora were revised, including GENETAG corpus (46), Gene Normalization corpus of BioCreative II (41), GENIA corpus (47), Yapex corpus (48), JNLPBA corpus (49), MedTag corpus (50), ProSpecTome corpus (51) and PennBioIE corpus (52). As with the CEM guidelines, the refined was done through an iterative process based on the annotation of sample by several annotators in parallel.

The annotated GPROs comprehended names, specific classes or fragments of genes/proteins/RNAs. Then, general concepts (isolated terms like ‘gene’, ‘receptors’, ‘proteins’, ‘mRNA’, ‘peptide’, ‘sequence’, ‘transcript’, ‘gene product’, ‘domain’ and ‘isolate’), lipids and small organic molecules are excluded from the annotation task.

In the annotation process, eight types of GPRO mention were differentiated, and their annotation was exhaustive. Mentions not included in those classes were not annotated. The DrugProt gene mention GSs do not include the GPRO classes. These eight classes were grouped into two types:

- GPRO entity mention type 1: covering those GPRO mentions that can be normalized to a bio-entity database record. GPRO mentions of this group appear in the GS as GENE-Y.
- GPRO entity mention type 2: covering those GPRO mentions that in principle cannot be normalized to a unique bio-entity database record. GPRO mentions of this group appear in the GS as GENE-N.

The annotation process required a large domain background knowledge and usage of specialized resources. Then, to obtain correct, high-quality annotations, the curators had an academic training in biology (molecular biology and genetics) or biochemistry.

#### DrugProt relation GS

The corpus comprises binary relation annotations between CEM and GPRO entities. During the annotation process, annotators were presented with abstracts containing entity mentions and were asked to mark the binary relation between them following the DrugProt relation annotation guidelines (https://doi.org/10.5281/zenodo.4957137). These guidelines provide curation rules to evaluate if a sentence within an abstract is describing a CEM–GPRO interaction and also include definitions to assign each identified interaction to any of the five classes and 16 subclasses of the corpus. The relation annotation guidelines were previously employed in the ChemProt–BioCreative VI task with a smaller corpus. These guidelines were refined after iterative cycles of annotations of sample documents, incorporating curators’ suggestions and solving annotation inconsistencies encountered when comparing results from different human curators.

**Figure 3.**
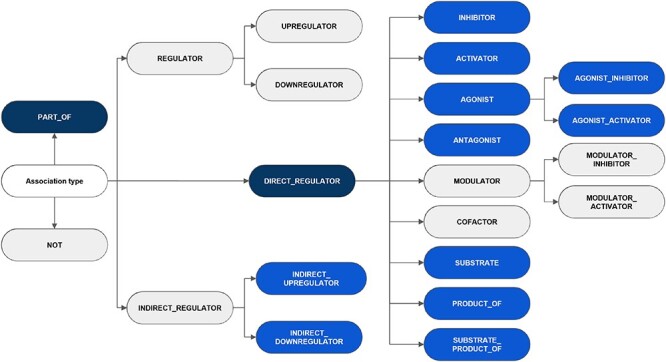
An overview of the hierarchy of DrugProt relation types and classification considered in annotation guidelines. The elements in blue represent those chosen for the DrugProt task, with dark-blue indicating the classes and light-blue representing the subclasses. They were selected based on their impact, the number of annotated instances, the internal consistency of the relation tree and the prediction performance determined by a baseline system (37).

It is noteworthy that although the annotation adhered to the five classes and 16 subclasses as defined in the guidelines, the low frequency of particular categories within the training set prompted the decision to release relations for two classes and 11 subclasses (a total of 13 relation types). The exhaustive list of classes and subclasses considered in the guidelines is shown in Figure 3, indicating the categories appearing in the published corpus. Other possible relations between CEMs and GPROs, such as phenotypic and biological responses, should not be labeled. Besides, the interactions were defined following the concept ‘what a CEM does to a GPRO’ (CEM $\rightarrow$ GPRO direction) and not the opposite direction (GPRO $\rightarrow$ CEM direction) (‘what a GPRO does to a CEM’).

To ensure a consistent nomenclature and to prevent redundancy in defining the relation classes, a review of various resources was conducted. These resources include chemical repositories that integrate chemical-biology information, such as DrugBank (38, 39), the Therapeutic Targets Database (53) and ChEMBL (54). In addition, the assessment took into account the BioAssay Ontology (BAO) (55), pre-existing formalizations for the annotation of relations like the biological expression language (BEL) developed for Track 4 of the BioCreative challenge (56), curation guidelines for transcription regulation interactions (DNA-binding transcription factor–target gene interaction) and SIGNOR, a database of causal relations between biological entities (57).

These resources were particularly important for different branches of the relation trees. For instance, for the set-up of the direct-regulator subclasses, SIGNOR, ChEMBL, BAO and DrugBank resources played a key role. For the indirect regulator subclasses, BEL, curation guidelines for transcription regulation interactions and SIGNOR were more relevant. In particular, BEL defines five classes of casual relations between a subject and an object term, which heavily influenced the relationship structure of indirect regulations. Additionally, the UPHAR/BPS Guide to Pharmacology in 2016 (58) determined the subclasses related to pharmacological modes or action.

The annotation process required extensive domain background knowledge. Annotators had an academic training in chemistry, biology (including molecular biology and genetics) and biochemistry. Moreover, their expertise extended to areas such as medicinal chemistry and pharmacology, ensuring the accuracy and high quality of the annotations. Regarding the IAA, the increased number of annotators posed challenges in calculating traditional IAA metrics. Therefore, a cross-validation process was employed, in which a subset of the documents was validated by a second, more experienced annotator.

### DrugProt corpus format

The DrugProt corpus is comprised of three fundamental components: the PubMed abstracts, the entity annotations (categorized as CEM and GPRO) and the relation annotations.

The PubMed abstracts are presented in a raw text format and are encoded using UTF-8. These abstracts are organized within a tab-separated text file, containing three distinct columns: the article identifier (referred to as PMID or PubMed identifier), the title of the article and the article’s abstract itself.

**Figure 4.**
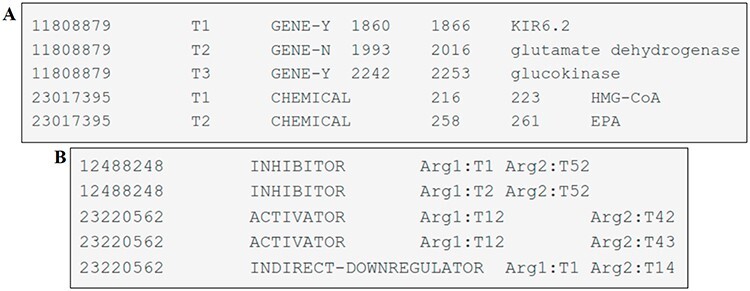
Examples of DrugProt (A) entity annotation and (B) relation annotation.

The entity mentions are provided in a tab-separated file with six columns. These columns have the following information: the article identifier (PMID), a term number (pertinent to the given record), the type of entity mention (CHEMICAL, GENE-Y and GENE-N), the start-offset (indicating the index of the first character of the annotated span in the text), the end-offset (the index of the first character after the annotated span) and the text span of the annotation. Each individual line in the file corresponds to an entity uniquely identified by its PMID and the term number. An example of one file can be seen in Figure 4A.

The file containing relation annotations consists of columns separated by tabs representing the article identifier (PMID), the DrugProt relation type, the relation argument 1 (of type CHEMICAL) and the relation argument 2 (of type GENE). Each line within this file represents a relation, and each relation is identified by the PMID, the relation type and the two related entities, as shown in Figure 4B.

For the DrugProt shared task, the DrugProt corpus was partitioned into three distinct subsets: training (consisting of 3500 abstracts), validation (comprising 750 abstracts) and test (also containing 750 abstracts). The split was random, while ensuring that all abstracts released during the prior ChemProt task were included within either the training or validation subsets. The DrugProt corpus is available on Zenodo (https://doi.org/10.5281/zenodo.4955410).

### DrugProt Large Scale corpus generation

Given the substantial costs linked to the manual generation of annotated datasets, previous research has explored alternative strategies, such as the previously mentioned silver standard corpora. The CALBC project (14) annotated 150 000 Medline abstracts using five automatic systems with different coverage and purposes. These systems were based on terminological resources rather than real-world data, and due to the absence of a pre-existing GS for system evaluation, the quality of the silver standard was unclear.

To address these challenges, the DrugProt Large Scale corpus was created, encompassing 2 366 081 English PubMed abstracts, including titles, with annotated CEM and GPRO entities. This corpus was developed through a document selection process that combined MeSH queries, outcomes of NER systems applied across the entire PubMed database, document classifiers’ results and database metadata, guided by 10 specific criteria. These 10 selection criteria were tailored to aggregate pertinent abstracts covering several domains, including gene expression, pharmacological action, viral zoonoses, rare diseases and coronavirus, all of which featured mentions of CEM–GPRO interactions. The full description of the document selection criteria is available on Zenodo (https://doi.org/10.5281/zenodo.5656991).

This document selection criterion allows having a large-scale corpus useful for many purposes including drug discovery, repurposing, design and metabolism, as well as for exploring drug-induced adverse reactions and off-target interactions, among other topics. The abstracts were downloaded on 17 June 2021 using the PubMed Bio.Entrez package. The pipeline used to download the abstracts is stored at GitHub (https://github.com/tonifuc3m/pubmed-parser).

The mention annotations were generated by running an NER tagger that adds context after the sentences to be tagged as this is shown to increase tagging performance (6). Also, for NER tagging the GENE-N and GENE-Y mentions were both converted to plain GENE mentions. This simplification was adopted to avoid unnecessary complexities in predicting which gene mentions can be normalized and which cannot. The NER tagger was evaluated on the test set of the DrugProt mention GSs. The trained NER models selected for large corpus tagging obtained a 92.38 exact match *F*1-score on the CEM mentions and a 90.34 exact match *F*1-score on the GPRO mentions.

The DrugProt Large Scale corpus consists of all abstracts selected on the basis of the aforementioned criteria, as long as they contain at least one CEM and one GPRO mention. The corpus has the same format as the DrugProt corpus (excluding the relation annotations) and it is available on Zenodo (https://doi.org/10.5281/zenodo.5119878).

### DrugProt shared task description

The BioCreAtIvE (Critical Assessment of Information Extraction systems in Biology) challenge evaluation consists of a community-wide effort for evaluating text mining and information extraction systems applied to the biological domain. Specifically, the DrugProt–BioCreative VII challenge evaluates systems that extract relations between chemical compounds or drugs and genes, proteins or miRNA in biomedical literature. In the shared task context, several resources have been developed: the DrugProt corpus (that contains three GSs), the DrugProt Large Scale corpus (containing two silver standard corpora), the official evaluation script (specifically developed for a unified evaluation of participating systems), a CodaLab evaluation page and two baseline systems.

Participants were asked to develop models for two separate subtasks: the Main DrugProt (DrugProt-M) track , which focuses on evaluating ER systems with high predictive performance, and the Large Scale DrugProt (DrugProt-L) Track , where participants’ systems are required to process large volumes of data volumes. The main goal of DrugProt-L is to evaluate participants’ design strategies for efficiently handling large datasets and to examine how these strategies impact predictive performance.

The challenge comprises both a training and a test phase. During the training phase, participants use the DrugProt corpus to develop their ER systems by means of supervised techniques. Then, in the evaluation phase, participants apply their systems to predict relations within a collection of PubMed abstracts that have only mention annotations. These predictions will be evaluated later on the CodaLab evaluation page and compared with baselines.

**Figure 5.**
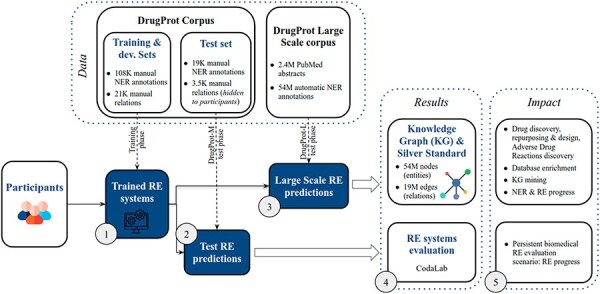
DrugProt shared task overview. Training phase (1), test phase (2,3), results (4) and impact (5).

DrugProt yields two main outcomes: first, participants’ large-scale predictions contribute to the creation of a comprehensive knowledge graph; second, their systems undergo evaluation, ensuring that future RE systems can also be assessed on the CodaLab platform. The overview of the challenge in terms of phases, tracks and exploitation of results is shown in Figure 5.

### Evaluation

During the training phase, all participants were given the abstracts, GPRO, CEM and relation manual annotations for the 4250 documents of the training and development sets.

During the test phase, DrugProt-M track participants received as well the abstracts, GPRO and CEM annotations of a set of 10 750 documents (including the test set and 10 000 background documents). The test set has manual annotations, while the background documents have automatic entity annotations and are provided to prevent manual annotations by participating teams. DrugProt-M track participants must return their automatic predicted relations for the 10 750 documents—five prediction runs are allowed per participating team. Finally, the predicted relations of the test set documents are compared against the manual, GS relations.

On the other hand, during the test phase, DrugProt-L track participants needed to make predictions for 2 366 081 documents, including the 750 test set documents.

The official evaluation metrics are micro-averaged precision, recall and *F*1-score. Due to the particular impact of the different relation types, detailed granular results by relation type, computed with the official evaluation kit, are provided as well.

All relations are binary and have three components: a CEM, a GPRO and a relation type. However, it is possible for a given CEM–GPRO pair to have more than one valid relation, although this situation is uncommon in the test set. In this case, all valid relation types must be predicted and they are evaluated independently.

The evaluation script is available on GitHub (https://github.com/tonifuc3m/drugprot-evaluation-library). In addition, the DrugProt-M track setting is maintained on CodaLab, and therefore future teams can be evaluated on the same conditions as original shared task participants.

### Baseline systems

To compare with the participants’ systems, two baselines are proposed. The first baseline is a maximum-recall system that considers every sentence co-mention of CEMs and GPROs as a positive relation. All possible relation types are assigned to each co-mention.

The second baseline, called the ‘Turku-BSC system’, is obtained by an RE system that we developed for the DrugProt challenge. The system is similar to the RE systems developed by Mehryary *et al.* (37) and utilizes a pretrained BERT transformer (a bioBERT-base model, retrievable from https://huggingface.co/dmis-lab/biobert-v1.1/tree/main) for encoding the input texts, along with a single decision layer with softmax activation for classification. In contrast to many previous RE systems that focus on a single sentence at a time (and thus fail to predict any cross-sentence relations), we allow ML examples to be generated even if the two entities (a CEM and a GPRO) are located in different sentences. This allows us to train with and extract both inner-sentence and cross-sentence relations. More specifically, we generate an example for two candidate-named entities, if the two mentions and the words before, after and between them can fit into a window of 128 BERT tokens. The window size is one of the optimized hyper-parameters and it directly affects the prediction performance, as well as the number of generated examples. Since an input text can include more than one CEM and/or GPRO entities, and because the RE task is performed similarly to a text classification task, we mark the beginning and end of entities of focus using unused tokens in the BERT vocabulary (e.g. [unused1]insulin[unused2]). We have previously shown that this marking approach slightly outperforms the masking approach (i.e. replacing entity names with predefined placeholders) (37). Finally, the system is optimized using a grid search to find optimal values for hyper-parameters including window size, learning rate, mini-batch size and number of training epochs. This is done by cycles of training the system on the training set with a set of hyper-parameters, predicting the development set and evaluating its performance.

## Results

### DrugProt corpus

The DrugProt corpus contains manually annotated mentions of CEMs, GPROs and the binary interactions existing between them. This corpus is provided together with annotation guidelines. It is relevant for developing gene (GPRO) and drug (CEM) recognition systems, as well as CEM–GPRO RE systems. In addition, data curators and the non-English NLP community seeking adaptation to other languages can benefit from this resource, among other potential user groups.

Table 1 presents an overview of the DrugProt corpus. It contains 24 526 manually annotated relations, divided into 13 relation types of biological significance, 61 775 manual GPRO entity annotations and 65 561 manual CEM entity annotations. This means that the gene and chemical mention GSs are among the largest manually annotated entity corpora in the biological domain and offer an opportunity to develop and evaluate the better NER systems.

**Table 1.** DrugProt GS statistics

		Training	Development	Test	Total
Abstracts		3500	750	750	5000
Relations	antagonist	972	218	154	1344
	agonist	658	131	101	890
	agonist-activator	29	10	0	39
	agonist-inhibitor	13	2	3	18
	direct-regulator	2247	458	429	3134
	activator	1428	246	334	2008
	inhibitor	5388	1150	1051	7589
	indirect-downregulator	1329	332	304	1965
	indirect-upregulator	1378	302	277	1957
	part-of	885	257	228	1370
	product-of	920	158	181	1259
	substrate	2003	494	419	2916
	substrate_product-of	24	3	10	37
	Total	17 274	3761	3491	24 526
Entities	gene	43 255	9005	9515	61 775
	chemical	46 274	9853	9434	65 561
	Total	89 529	18 858	18 949	127 336

**Figure 6.**
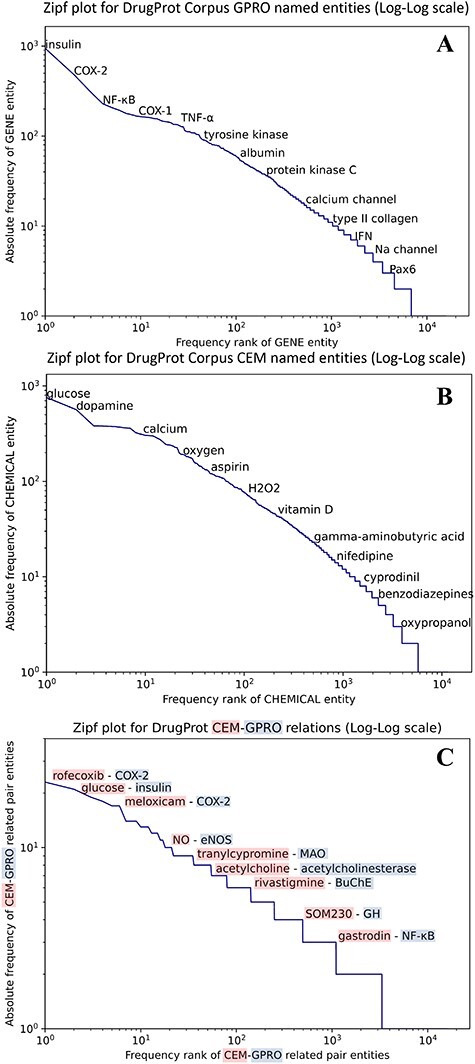
(A) Zipfs plot of all DrugProt GPRO entities, (B) all DrugProt CEM entities from GS and (C) Zipf plot of CEM–GPRO related pairs in the DrugProt corpus.

We carried out an analysis to provide an overview of the content of the DrugProt corpus. Figure 6 shows (A) the statistical profile of the CEM entities and (B) the GPRO entities present in the corpus by examining the mention distribution. It reflects the typical behavior of the token frequencies in a corpus. Most CEMs and GPROs in the DrugProt corpus have a low frequency. Indeed, 71.1% of GPROs has a frequency of 1 or 2, and the percentage for CEMs is 65.4%. Additionally, CEM mentions tend to be longer than GPRO ones: the longest GPRO mention has 105 characters, and the median length is 6. The longest CEM mention has 174 characters, and the median is 9.

We have also analyzed the overlap between both mention types. This can happen, since the mention annotation was independent for the two entity types. In total, 659 mentions are annotated as CEM and GPRO. Some overlapping entities are ‘angiotensin’, ‘oxytocin’, ‘Ang II’ (an abbreviation of angiotensinII), ‘GnRH’, ‘vasopressin’, ‘AVP’, ‘somatostatin’ and ‘bradykinin’.

In the case of the DrugProt relations, the distribution of CEM–GPRO relation pairs follows the expected pattern of token frequency distribution in a corpus, as shown in Figure 6C. The majority of entities exhibit low frequencies, with 94.2% of CEM–GPRO pairs having frequencies of 1 or 2. Conversely, a small subset of pairs is more prevalent. For a more detailed insight, tables presenting the most frequent pairs are provided in the Supplementary material.

**Figure 7.**
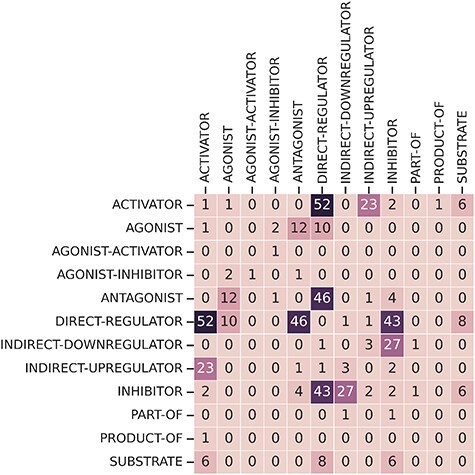
Frequency overlap between different relation types.

Finally, some relations have multiple relation types. There are 249 CEM–GPRO pairs with two annotated relation labels. Figure 7 shows that the relation types that overlap the most are activator and direct-regulator, followed by antagonist and direct-regulator. The DrugProt corpus is available on Zenodo (https://doi.org/10.5281/zenodo.4955410).

### DrugProt Large Scale corpus

Real-world applications in NLP often demand the processing of extensive and diverse datasets. Therefore, the development of scalable pipelines that can handle big collections of documents is crucial. This issue is particularly relevant in clinical applications, where the lack of scalability has been identified as a barrier to NLP progress (5).

Nonetheless, biomedical NLP challenges and shared tasks frequently provide corpora of limited or moderate sizes, typically focused on specific subdomains, primarily due to the significant expenses linked to the manual creation of annotated datasets. To overcome this limitation, silver standards have been introduced, enabling the training of models with improved performance across several tasks (41, 59, 60).

The DrugProt Large Scale corpus contains automatically annotated mentions of CEMs and GPROs in 2 366 081 PubMed abstracts. Then, it is directly relevant to the development of NER pipelines. Besides, the DrugProt Large Scale corpus is distributed as part of the Large Scale DrugProt track. Participants of this competition must generate relation predictions in this large, heterogeneous set of documents. The goal here is 3-fold: first, to assess whether RE pipelines are capable of scaling up to process large literature volumes; second, to compare the prediction performance difference between scalable and non-scalable systems; finally, to merge the participants’ relation predictions and generate a knowledge graph useful for different topics, covered in the large-scale corpus document selection (drug discovery, drug design, off-target interactions, etc). A description of the generated knowledge graph and the potential uses is presented in the section ‘DrugProt Large Scale Silver Standard Knowledge Graph’.

For the generation of the DrugProt Large Scale corpus, 3 966 792 PubMed abstracts were selected according to the document selection criteria. After filtering out the documents with an empty title, or empty abstract body, or that did not have at least one sentence with a GPRO and a CEM, the remaining number of PubMed abstracts is 2 366 081. In them, there are 33,578,479 GPRO and 20 415 123 CEM mentions. Table 2 contains the overview statistics of the DrugProt Large Scale corpus.

**Table 2.** General statistics of the DrugProt Large Scale dataset provided to participants

		Number of entities		
Subset	Number of documents	GPRO	CEM	Total
Background set of DrugProt-M track	10 000	157 523	134 333	291 856
Original PubMed dump	3 966 792	47 622 872	22 993 509	70 616 381
DrugProt Large Scale corpus	2 366 081	33 578 479	20 415 123	53 993 602

**Figure 8.**
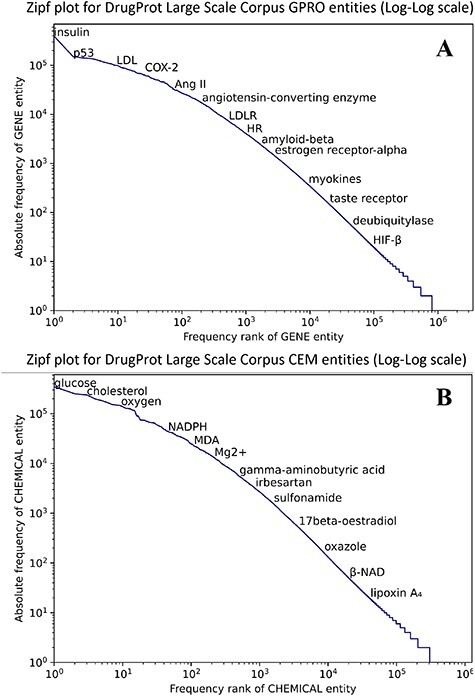
Zipf plot of GPRO (A) and CEM (B) entities in the DrugProt Large Scale corpus.

Figure 8 shows that both CEM and GPRO entities within the silver standard follow the typical behavior in terms of the token frequency of a corpus. Although a small number of entities are prominently represented, the majority of GPRO mentions (73.2%) occur only once or twice, and similarly, the majority of CEM mentions (71.6%) have a frequency of 1 or 2.

In terms of mention length, it is remarkable that CEM mentions are notably longer than GPRO mentions, a pattern consistent with the GSs. While the longest GPRO mention spans 155 characters with a median length of 5, the longest CEM mention extends to 508 characters, with a median length of 8. The DrugProt Large Scale corpus is available on Zenodo (https://doi.org/10.5281/zenodo.4955410).

### DrugProt Large Scale Silver Standard Knowledge Graph

DrugProt offers a unique opportunity for the creation of a large and high-quality silver standard knowledge graph focused on CEM–GPRO relations. This resource is created through the large amount of data within the DrugProt Large Scale corpus, incorporating CEM and GPRO entity predictions from both the silver standard and the large-scale track participants. It constitutes an enormous, high-quality resource of automatically annotated CEM–GPRO relations. Each relation is paired with a corresponding precision score, meticulously calculated using DrugProt’s GS test set. This feature empowers the capacity to selectively filter or intelligently merge diverse relation predictions, enhancing the precision and customization of the knowledge graph’s insights.

The DrugProt Large Scale Silver Standard Knowledge Graph is a relevant resource for RE system developers: it constitutes an extensive training and evaluation dataset. It can also significantly impact the data curator community since it is a valuable starting point to generate manual CEM–GPRO annotations with minimum effort; and the database community because it is ready to be consumed by biological databases.

The information in the DrugProt Large Scale Silver Standard Knowledge Graph is ready to be used as a knowledge graph. In the graph, the CEM and GPRO entities are nodes and the predicted relations are edges. Every node has a unique weight based on the combination of the micro-average precisions of every system that predicted the edge. We foresee its impact to explore the CEM–GPRO relations described in the literature or to predict novel chemical–gene interactions, among other uses.

This knowledge graph is a weighted bipartite graph since it has two types of nodes, CEM and GPRO; directed because relations go from CEM to GPRO; and with 13 types of edges, one per relation type.

In addition to this ready-to-use knowledge graph, the information stored in this resource allows the creation of subgraphs per relation type. Also, we suggest the creation of another knowledge graph in which the nodes are PubMed records, and two PubMed records are connected with an edge if they share a CEM or GPRO entity. The nodes would have the assigned MeSH terms as node attributes. To this extent, we have released the list of MeSH terms per each PubMedID included in the DrugProt Silver Standard Knowledge Graph.

As a showcase, we analyze the antagonist subgraph. It is a sparse graph with 95 812 nodes and 274 401 edges. Indeed, the clustering coefficient is 0.04, and the transitivity is 0.01. The graph is dissortative, as biological networks tend to be, being the assortativity degree −0.1. It has one giant component with 92 267 nodes, and 1478 disconnected components with a few nodes (mostly, 2 or 3) and a diameter of 15.

**Figure 9.**
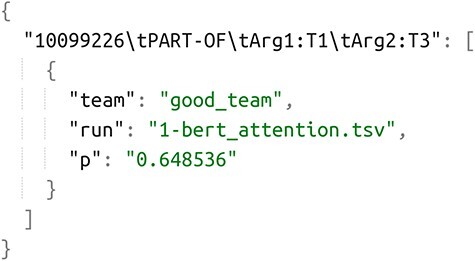
Example of JSON annotation of DrugProt Silver Standard corpus.

The Silver Standard Knowledge Graph format follows a similar structure as all other DrugProt corpora. Indeed, the abstracts and entity files are the DrugProt Large Scale corpus. However, the relation annotations are stored in JSON files with the structure shown in Figure 9. Each abstract is represented by a JSON file, with relation annotations following the same tab-separated format as the DrugProt GS. These annotations serve as keys, while the corresponding values are arrays of predictions denoted by ‘team’, ‘run’ and ‘p’ (micro-average precision on the test set for that specific run).

The complete DrugProt Silver Standard Knowledge Graph contains 53 993 602 nodes (CEM and GPRO entities) and 19 367 406 edges (unique CEM–GPRO relation predictions). In total, there are 146 864 121 predictions. Then, on average, every relation has 7.58 individual predictions. The average degree coefficient is 0.71 and the networks are highly sparse. Table 3 contains the number of predictions and knowledge graph edges per relation type.

**Table 3.** DrugProt Silver Standard Knowledge Graph overview statistics

Relation type	Predictions	Unique predictions (knowledge graph edges)
antagonist	4 597 943	533 536
agonist	3 315 925	463 888
agonist-activator	25 596	6468
agonist-inhibitor	36 171	4683
direct-regulator	13 572 608	2 425 063
activator	13 727 350	2 034 690
inhibitor	34 863 037	3 636 934
indirect-downregulator	18 066 689	1 882 009
indirect-upregulator	19 091 394	2 277 607
part-of	17 711 521	2 174 233
product-of	7 341 295	1 248 229
substrate	14 497 706	2 668 567
substrate_product-of	16 886	11 499
Total	146 864 121	19 367 406

The DrugProt Large Scale Silver Standard Knowledge Graph is available on Zenodo (https://doi.org/10.5281/zenodo.7252201). As an additional resource, we used the DrugProt NER Taggers and the Turku-BSC RE system to generate GPRO, CEM and relation annotations for the full PubMed dump from December 2021 (https://zenodo.org/record/7 252 238).

### Shared task participation overview

The task impact in terms of participation has been significant. A total of 30 teams, comprising 110 individuals, submitted 107 runs for the DrugProt-M track, while 9 teams submitted 21 runs for the DrugProt-L track. This level of engagement represents the highest participation observed in a BioCreative task to date. A summarized breakdown of the participating teams is shown in Table 4, including the tasks they contributed results to and links to associated software when available, please refer to Table 4.

**Table 4.** DrugProt team overview

ID	Team Name	Affiliation	Country	Tasks	Ref.	Tool URL
15	Humboldt	Humboldt-Universität	Germany	M	(61)	(62)
18	NLM-NCBI	National Institutes of Health	USA	M/L	(63)	–
13	KU-AZ	Korea University, AstraZeneca, AIGEN Sciences	South Korea, UK	M/L	(64)	–
7	UTHealth-CCB	University of Texas	USA	M/L	(65)	–
21	bibliome	INRAE	France	M	(66)	(67)
3	CU-UD	University of Delaware	USA	M/L	(68)	(69)
29	TTI-COIN	Toyota Technological Institute	Japan	M	(70)	-
4	good team	Guangdong University of Foreign Studies	China	M/	–	–
23	FSU2021	Florida State University	USA	M/L	(71)	(72)
14	HY-NLP	Hanyang University	South Korea	M	–	–
28	NVhealthNLP	NVIDIA	USA	M/L	(73)	(74)
16	HITSZ-ICRC	Harbin Institute of Technology	China	M	(75)	–
6	Saama Research	Saama Technologies	India	M	–	–
10	Stelios	–	Greece	M	–	–
5	The Three Musketeers	Fudan University	China	M/L	–	–
2	USMBA_UIT	Sidi Mohamed Ben Abdellah University	Morocco	M	(76)	(77)
19	NLPatVCU	Virginia Commonwealth University	USA	M	(78)	(79)
27	BIT.UA	University of Aveiro	Portugal	M	(80)	–
25	Jungfraujoch	University of Zurich & ETH Zurich	Switzerland	M	–	(81)
24	CLaC	Concordia University	Canada	M	(82)	–
26	catalytic	Catalytic DS, Inc.	United States	M	(83)	–
8	DigiLab-UG	University of Geneva	Switzerland	M	(84)	–
1	Trerotola	University of Brescia	Italy	M	–	–
17	BHAM	University of Birmingham	UK	M	–	-
11	LasigeBioTM	LASIGE	Portugal	M	(85)	(86)
9	TMU_NLP	Taipei Medical University	Taiwan	M/L	(87)	–
12	Elsevier Health D.S.	Elsevier	USA	M	–	–
20	Orpailleur	Université de Lorraine, CNRS	France	M	–	(88)
30	NetPharMed	University of Helsinki	Finland	M	(89)	–
22	CanSa	Al Baha University	Saudi Arabia	M	–	–

A/I stands for academic or industry institution. In the Tasks column, M stands for the Main DrugProt track and L for the Large Scale DrugProt track. The teams are sorted based on their performance in the M-track.

### Evaluation results

In the DrugProt-M track, the outcomes achieved by all teams are presented in Table 5. The Humboldt team obtained the top-scoring results with a micro-average *F*1-score of 0.797311. In a run, they also obtained the highest micro-average precision (0.815075). On the other hand, the FSU2021 team achieved the highest micro-average recall, reaching a score of 0.824355.

**Table 5.** Best run results of the DrugProt-M track.

ID	Team	Run	Precision	Recall	*F1-score*
15	Humboldt	1	0.7961	0.7986	**0.7973**
18	NLM-NCBI	5	0.7847	0.8052	0.7948
13	KU-AZ	2	0.7972	0.7817	0.7894
7	UTHealth	2	**0.8044**	0.7496	0.776
21	bibliome	2	0.7546	0.7966	0.775
3	CU-UD	3	0.7709	0.7771	0.774
29	TTI-COIN	1	0.7493	0.7776	0.7632
4	good team	5	0.7344	0.794	0.763
23	FSU2021	4	0.754	0.751	0.7525
14	HY-NLP	1	0.7122	0.792	0.75
28	NVhealthNLP	4	0.7732	0.7249	0.7483
16	HITSZ-ICRC	4	0.7671	0.7183	0.7419
6	Saama Research	1	0.7406	0.7361	0.7383
10	Stelios	4	0.7315	0.7261	0.7288
5	The Three Musketeers	1	0.6993	0.7564	0.7268
2	USMBA_UIT	4	0.7569	0.6745	0.7133
19	NLPatVCU	1	0.7335	0.6908	0.7115
27	BIT.UA	2	0.7003	0.7229	0.7114
25	Jungfraujoch	1	0.7798	0.6201	0.6908
24	ClaC	3	0.6444	0.7014	0.6717
26	catalytic	1	0.6746	0.5822	0.625
8	DigiLab-UG	4	0.4507	**0.8794**	0.5959
1	Trerotola	1	0.3149	0.8378	0.4578
17	BHAM	1	0.2305	0.3673	0.2833
11	LasigeBioTM	1	0.369	0.1865	0.2478
9	TMU_NLP	2	0.5678	0.1224	0.2013
12	Elsevier	1	0.5947	0.0576	0.105
20	Orpailleur	3	0.3078	0.0438	0.0767
30	NetPharMed	1	0.0395	0.1573	0.0631
22	Cansa	1	0.0	0.0	0.0
	Max-recall baseline	1	0.0022	1.0	0.0044
	Turku-BSC system	1	0.755	0.734	0.744

Best results bolded.

Table 6 presents the DrugProt-L track results. This task aimed to analyze whether RE systems could maintain high performance while processing large volumes of input data. The results indicate that this is indeed the case, as the performance discrepancies between the Large Scale and Main DrugProt tracks are minimal. For instance, the NLM-NCBI team achieved the highest micro-average *F*1-score of 0.788602 in the Large Scale DrugProt track, while they obtained 0.794796 in the Main DrugProt track.

**Table 6.** Large Scale DrugProt track results

ID	Team	Run	Precision	Recall	*F1-score*
18	NLM-NCBI	1	0.778186	0.789112	0.783611
		2	0.772977	0.804871	**0.788602**
		3	0.775049	0.795702	0.78524
		4	0.767621	0.798854	0.782926
		5	0.747794	0.825788	0.784858
13	KU-AZ	1	0.760092	0.755301	0.757689
		2	0.764415	0.752149	0.758232
		3	0.767303	0.736963	0.751827
7	UTHealth-CCC	1	0.763804	0.713467	0.737778
		2	0.77619	0.747278	0.76146
		3	0.794856	0.752722	0.773216
		4	**0.800799**	0.746418	0.772653
		5	0.797194	0.748997	0.772345
3	CU-UD	1	0.746575	0.780802	0.763305
4	good team	1	0.720129	0.766762	0.742714
23	FSU2021	1	0.70657	0.727221	0.716747
28	NVhealthNLP	1	0.732492	0.332665	0.457537
9	TMU_NLP	1	0.432432	**0.848138**	0.572811
		2	0.450187	0.828653	0.583417
		3	0.437236	0.799427	0.565292
5	The Three Musketeers	1	0.693691	0.58596	0.63529
	Max-recall baseline	1	0.0022	1.0	0.0044
	Turku-BSC system	1	0.755	0.734	0.744

Best results bolded.

Although the DrugProt-L track was not focused on analyzing prediction times, but on assessing the feasibility of adapting the models to handle large volumes of data without experiencing a significant decrease in performance, many participants have reported their prediction times. Reported times vary based on available computational resources, ranging from 9 h reported by KU-AZ using 16 GPUs concurrently, to 40 h reported by TMU_NLP, 53.5 h reported by UTHealth and even 5 days employed by NLP or FSU2021.

**Figure 10.**
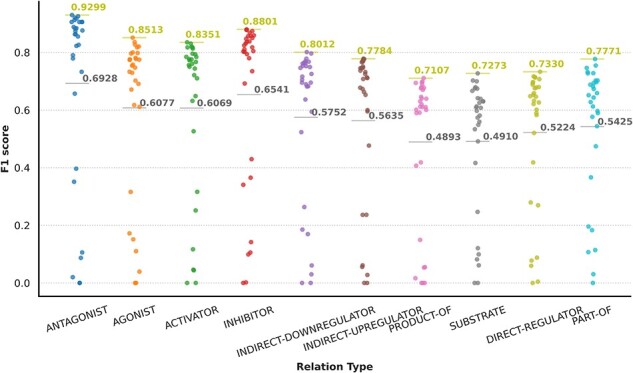
Graphical representation of participants’ results in granular format for the DrugProt-M track.

Analyzing system performance across different relation types is of great significance to align ER systems with their final applications. Figure 10 illustrates the participant’s *F*-score results for each of the corpus relation types. In the graph, each point represents the result of each run of the system participants. The top-performance team result is shown as a golden bar with a label, while the average value for each relation type is represented in gray. It is observed that the categories antagonist, inhibitor, agonist and activator exhibit more favorable average prediction results across all participants, with the best team in each category achieving performance over 0.83. The performance varies depending on the relation type, and those categories that had a very small number of samples in the test set have been excluded from the graph since the results are not completely representative. The detailed numerical results, including precision and recall values, can be found in the Supplementary material of this publication.

### Participating systems—methodological analysis

DrugProt participants generally treat the RE problem as a sentence classification task. The most common pipeline for generating a CEM–GPRO relation prediction is to (i) split the input text into sentences, (ii) select those sentences that contain a marked CEM and a marked GPRO, (iii) tokenize the sentence into corresponding tokens (in general, subwords), (iv) pass the tokens to a transformer-based LM and (v) input the first output of the transformer (the [CLS] token) into a simple classifier. The classifier would then return either a negative prediction (no relation is detected) or categorize the relation into one of the 13 DrugProt relation types.

Several modifications to this common pipeline are frequently employed by participants, and some of the most significant ones are detailed below:

Knowledge base information: teams that integrated knowledge bases in the information encoding step reported an increase in performance (61,70).

**Figure 11.**
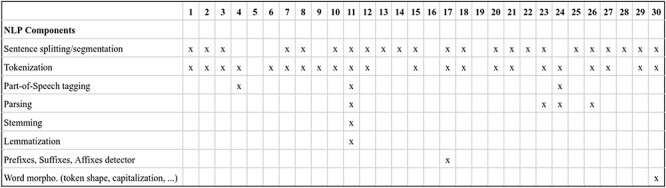
An overview of NLP components used by DrugProt participants: there is no information about team 16.

NLP components: beyond sentence splitting and tokenization, there is a rich diversity in the NLP components used by DrugProt participants. It is exciting to observe the divergence in the treatment of the named entities. Before passing the tokens to the LM, it is common to substitute the CEM and GPRO entities with standard tokens (masking) or add flag tokens before and after them (marking). These techniques are intended to help the LM to identify the entities involved in the relation. Figure 11 contains an overview of the NLP components employed, including the entity masking/marking strategy.

**Figure 12.**
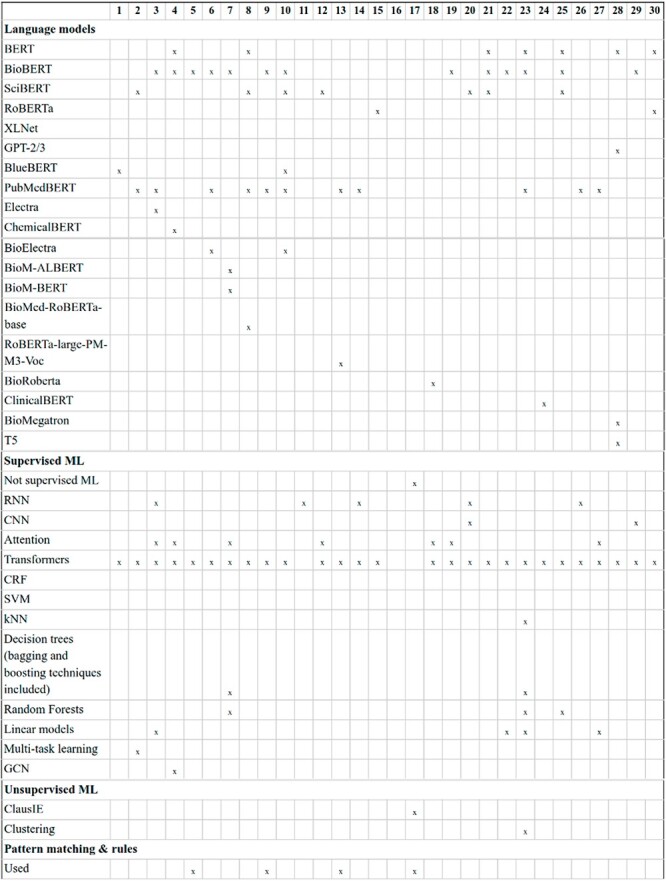
An overview of NLP systems used by DrugProt participants (part I): there is no information about team 16.

**Figure 13.**
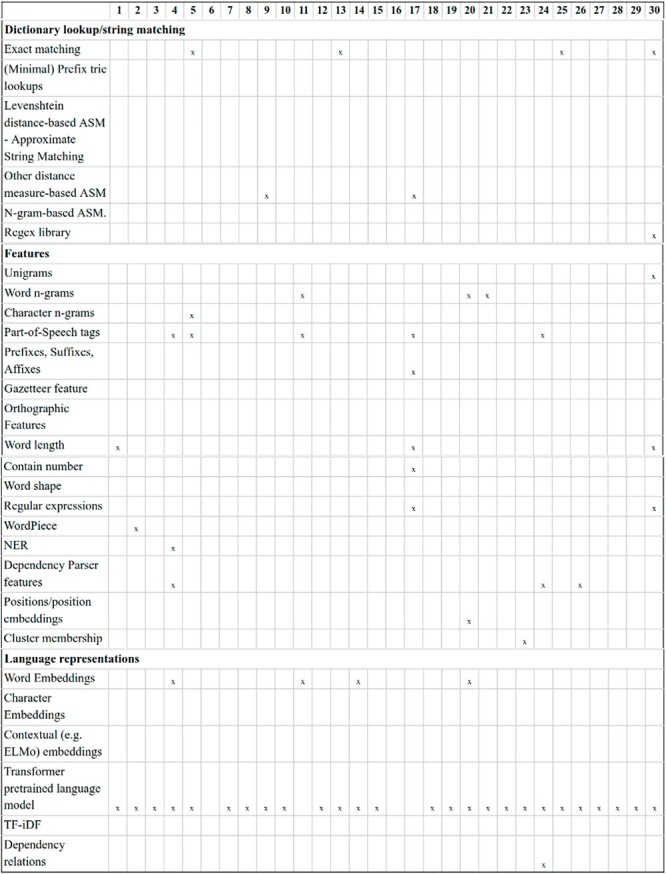
An overview of NLP systems used by DrugProt participants (part II): there is no information about team 16.

Transformer-based LMs: the diversity in transformer-based LMs experienced by the NLP community in recent years is evident in DrugProt. A common difference among participants consists of changing the LM, and many of them compared the performance variations (e.g. DigiLab-UG (84)). Figures 12 and 13 contain an overview of the system types used by the participants, with the different LM included, with BioBERT and PubMedBERT being the most popular ones.

Classifier layer: implementing a linear or a softmax classifier to categorize the sentence is common. The three best-performing teams in the task use this strategy, indeed. However, some exciting variations of this strategy include also attention, long short-term memory or CNN layers after the transformer output.

Post-processing: it is also common to include simple post-processing rules such as removing common false positives (FPs) by stopwords detection.

Ensemble: it is the most popular and impactful modification. A significant performance increase is detected by many DrugProt participants when ensembling different RE systems. The simplest ensemble scenario involves having the same architecture trained with *N* different hyper-parameter initializations. For prediction, the same sentence passes through all the models. Since we end up with *N* predictions for each sentence, a voting strategy is applied to get one single prediction. Majority voting is the most common voting strategy. Other, more complex ensemble scenarios include smart voting strategies based on clustering (FSU2021 (71)) or using a multilayer perceptron with a softmax layer to combine the different outputs (CU-UD (68)). However, these approaches did not overcome a majority voting strategy.

**Figure 14.**
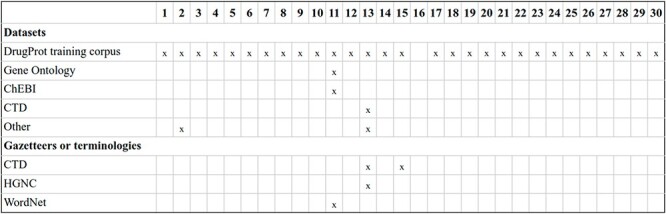
An overview of training information and datasets used by DrugProt participants: there is no information about team 16.

For RE system training, most participants opted for using only the DrugProt GS and varying the architecture or hyper-parameters. Figure 14 contains an overview of the training and input information employed by DrugProt participants.

Some noteworthy exceptions are the KU-AZ team, which generated silver standard predictions with an initial model and used it to retrain a larger model, and the USMBA_UIT team, which combined annotated datasets from different sources to create a pretrained model through multitask learning.

#### Model adaptation for large corpora

In terms of system adaptions for the DrugProt-L track, most teams opted for simpler architectures to accelerate the prediction process. They relied on more efficient pretrained models such as PubMedBERT or BioBERT, often using their ‘base’ versions and employing data-slicing strategies to distribute the computational load across multiple GPUs in parallel when available.

Among the participants, NLM-NCBI achieved the best performance results using an ensemble strategy similar to the one they used in DrugProt-M, but with standard losses for model weight adjustment. Conversely, the KU-AZ team chose not to use ensemble strategies due to their computational demands, opting instead for prediction with a single model and preprocessing beforehand. The TMU_NLP team implemented interesting candidate sentence selection strategies to improve the efficiency of their information extraction systems. Additionally, they incorporated vector distance-based features to identify potential words within sentences related to the relations that needed to be predicted.

### Participating systems—top 3 participants description

The system with the highest micro-*F*1 score was Humboldt and they also obtained the highest *F*1-score for direct-regulator, indirect-upregulator, inhibitor and product-of relation types. The authors defined the task as a sentence classification problem. The sentence was input to the biomedical pretrained transformer LMs RoBERTa-large-PM-M3-Voc. The classification was performed with a linear layer applied to the output of the transformer for the [CLS] token of the LM. Entity descriptions from the CTD database were used to enrich the model information. The best results were obtained by ensembling 10 models by averaging the predicted probabilities of every instance.

The NLM-NCBI team obtained the second-highest micro *F*1-score and the highest *F*1-score for the relation types antagonist, agonist, agonist-inhibitor, substrate and part_of. They tested two approaches to solving the challenge: text classification and sequence labeling. Again, biomedical pretrained LMs are used for both frameworks, including, but not only, PubMedBERT. On top of the LM, a softmax layer was applied to the output of the transformer for the [CLS] token to perform text classification. In contrast, for the sequence labeling approach, a fully connected layer and a softmax classification layer were applied to obtain predictions for each token. The best results were obtained by ensembling with the ‘majority voting’ strategy all the text classification and sequence labeling models.

Finally, the team KU-AZ obtained the third-highest micro *F*1-score and the highest *F*1-score for the relation types indirect-downregulator and agonist-inhibitor. They augmented the DrugProt dataset by predicting labels with transformer models and built a larger dataset refined with a knowledge base. Then, the challenge was modeled as a text classification task. Instances were passed through a biomedical pretrained LM, and a linear classification layer was applied to the output of the transformer for the [CLS] token. Finally, models were ensembled. The authors report that data augmentation has worked remarkably well for relation types with few examples.

### Error analysis of the participating systems

In this section, we have compared the participants’ predictions in the DrugProt-M track with the test set of the DrugProt corpus. We analyse two types of errors: false negatives (FN), where a relation is present in the DrugProt corpus but not in participants’ predictions, and FPs, where relations are predicted by the participants but not found in the DrugProt corpus. In particular, we have analyzed the three main aspects: (i) the entities involved in the FN and FP, (ii) the relation types attributed to the FN and FP and (iii) the balance between precision and recall for various relation types.

#### Most common entity errors

Table 7 presents some of the entities that are frequently associated with a higher number of prediction errors. Among these entities, several with the highest frequencies in the test set, such as ‘Ca^2+^’, ‘N’ or ‘COX-2’, are also frequently found in relations that were inaccurately predicted. This behavior is reasonable as their higher occurrence in the corpus might lead to a greater likelihood of errors. However, other entities like ‘sulindac’ and ‘BAY 50-4798’, which have lower frequencies in the corpus, also appear in the list of most common errors. This phenomenon can be attributed to the fact that mentions similar to those are linked with different relation categories in the training and test sets, posing a challenge for systems to generalize effectively. For instance, the mention ‘BAY 50-4798’ predominantly appears in activator and inhibitor relations in the training set, whereas it is associated with indirect-downregulator and indirect-upregulator relations in the test set. A similar trend is observed with the GPRO entities. For instance, the entity ‘cyclin D1’ is implicated in numerous FP and FN instances. In the training set, it predominantly participates in inhibitor relations, whereas in the test set, it is primarily associated with indirect-downregulator relations.

**Table 7.** List of the most frequent FNs and FPs in participants’ predictions

		FNs	FPs
CEM	Ca^2+^	1711	1339
	N	1216	1096
	BAY 50-4798	1077	921
	Sulindac	944	843
GPRO	COX-2	1873	2151
	AChE	1570	1468
	Cyclin D1	822	650

For a more extensive list, please refer to the Supplementary material.

#### Most common relation errors

**Figure 15.**
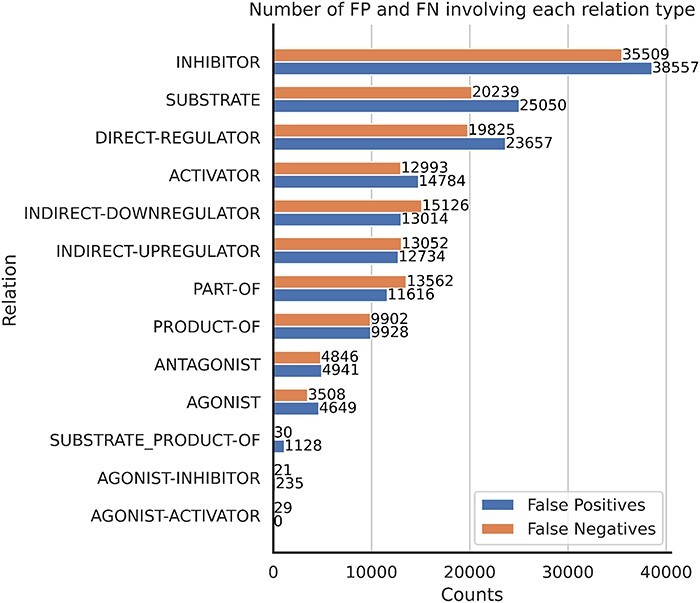
The number of FP and FN predictions for each relation type.

In terms of relation prediction errors, Figure 15 presents the distribution of errors across different relation types. Notably, the inhibitor, substrate and direct-regulator relations exhibit the highest number of FNs. Given that the inhibitor relation is the most prevalent within the corpus, it is possible that RE systems should have incorporated some downsampling mechanism to mitigate this effect. Conversely, relations like substrate and direct-regulator demonstrate lower recall rates (as shown in the Supplementary material), leading to an elevated FN rate.

#### The precision–recall balance varies per relation type

We can categorize the relation types into three distinct groups based on the balance between systems’ precision and recall. The first category consists of relation types where systems exhibit a balanced precision and recall, such as activator and inhibitor. The second category includes relation types where precision tends to be higher than recall, such as substrate, direct-regulator and agonist. The third category encompasses relation types where recall is the highest, including product-of, indirect-downregulator, indirect-upregulator and antagonist.

**Figure 16.**
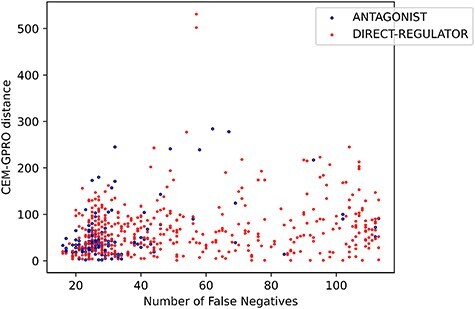
Relation between the FNs and the distance between the CEM and GPRO entities for antagonist and direct-regulator relations.

Relation types with a higher recall tend to have a relatively low FN rate, with FNs primarily influenced by the character distance between CEM and GPRO mentions. This phenomenon is represented in Figure 16, where the character distance between CEM and GPRO mentions in each prediction is plotted against the corresponding number of FNs. The antagonist relation, with a high recall in system predictions, shows a stronger correlation between FNs and the distance between mentions compared to lower recall relations like direct-regulator.

#### Overlapping mentions

Interestingly, the number of errors in overlapping mentions is remarkably low. An illustrative example of this scenario involves a GPRO entity labeled as ‘histidine triad’ and a corresponding CEM entity labeled as ‘histidine’. This suggests that the presence of overlapping mentions does not appear to significantly perplex modern transformer-based systems.

### Software analysis

**Figure 17.**
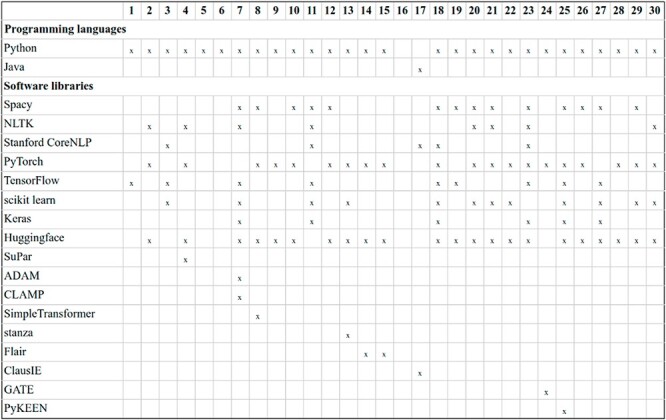
Description of the software used by DrugProt participants: there is no information about team 16.

Figure 17 summarizes the programming languages and software libraries employed by DrugProt participants. By far, Python is the most popular programming language. TensorFlow and Keras are employed by fewer teams than PyTorch for the deep learning Python libraries. For the NLP libraries, SpaCy is more popular than NLTK among participants. It is noteworthy the ubiquity of HuggingFace: among the participants using transformer-based LMs, only four do not report using the resources provided—and from those, two do not report any detailed software information.

## Discussion

The DrugProt shared task has considerably impacted the biomedical NLP community.

**Figure 18.**
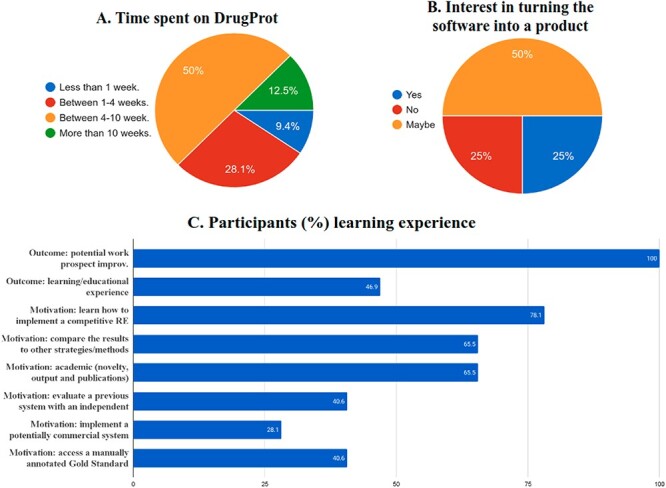
Survey results on the (A) time spent, (B), commercial interest and (C) motivation and outcomes of DrugProt participants.

First, it has impacted the participant institutions. Shared tasks help improve the state of the art and development resources. But they are also a powerful mechanism for training professionals and transferring knowledge from academia to industry. Figure 18 shows the time invested by DrugProt participants in the track according to their answers to a survey, and Figure 18C contains the motivation and learning experience outcomes of the survey. The figures provide relevant insight, considering that, despite 65% of participants reported having previous experience on RE, we have promoted that 35% of the people involved got introduced to the field. Besides, only 15% of the teams had previously worked on the generation of large-scale NLP systems, and there is a need to promote the development of scalable and robust pipelines. A part of the bottleneck may come from tasks usually focusing on optimizing systems for smaller datasets, and these initiatives are the front door for many groups. More effort on tasks focused on large-scale processing is needed.

One of the motivations for shared tasks is to promote open software development and to transfer knowledge from academia to industry. Indeed, from the survey answered by DrugProt participants, 75% of them would potentially be able to provide a software product out of their RE system (Figure 18B). As a summary, DrugProt, including the large-scale track, has impacted beyond traditional, purely academic evaluation scenarios. It has impacted participating teams regarding knowledge transfer to industry and knowledge discovery among others.

Second, it has been the BioCreative task with the most extensive participation: 107 people from 30 teams from four continents, including academia and industry. The developed systems offer high quality for most relations. For instance, 29 systems with an *F*1-score of >0.9 for antagonist relations exist. The systems are scalable to millions of documents. This was possible thanks to the latest-generation NLP systems based on transformers.

Third, it will maintain its impact over time. It has been a pioneering task. It was specifically designed to generate systems that can be readily applied to solve real-world problems. The relation-type definition, document selection criteria and evaluation scenario were designed following this idea. Besides, the Large Scale DrugProt track is the first one of its kind in the biomedical NLP community. However, there is still room for public evaluation of the systems, not only in terms of their predictive performance but also in terms of the time required to carry out these predictions. This requires the use of comparable evaluation environments, which provide equal computational capabilities for the participating models.

**Figure 19.**
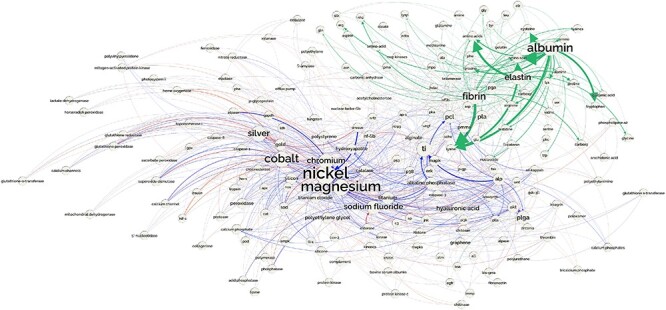
Network showing the part of the CEM–GPRO relations related to biomaterials extracted from the PubMed knowledge graph. part-of relations are shown in green, inhibitor in blue and activator in red.

The resources made available through DrugProt are expected to have a substancial impact on the biomedical NLP community. The DrugProt corpus, the DrugProt Silver Standard and the relation annotations for the entire PubMed include entity and relation types purposefully generated for a practical application in real-wold scenarios including drug discovery or drug repurposing, among others. The developed systems are powerful tools to complete the existing (or new) databases. However, more interaction with the curators’ communities is needed. For instance, DrugProt focuses on chemical–gene/protein interactions. But we could complete this knowledge with other relation types already studied such as protein–protein (15), chemical–chemical (90), gene–disease (18) or transcription factor–gene (91). The analysis of the generated knowledge graph could also foster research on biomaterial compounds. To illustrate its practical utility, Figure 19 shows a network generated by extracting the relations between entities related to the biomaterials domain, which could help to enrich existing resources in this field (92)

The DrugProt initiative presents opportunities for further exploration and development. One avenue involves the normalization of CEM and GPRO entities, addressing a task that remains incomplete in numerous biomedical NLP applications. Additionally, there is potential to extend the application of the developed systems to diverse data types, including full-text articles and patents. Furthermore, the incorporation of additional relation types not yet covered could also enhance the scope and utility of DrugProt’s contributions.

The developed systems are powerful tools to complete the existing (or new) databases. However, more interaction with the curators’ communities is needed. For instance, DrugProt focuses on chemical–gene/protein interactions. But we could complete this knowledge with other relation types already studied such as protein–protein (15), chemical–chemical (90), gene–disease (18) or transcription factor–gene (91).

Finally, DrugProt aims at generating persistent resources for the biomedical community. Then, the evaluation scenario is maintained intact on CodaLab (https://codalab.lisn.upsaclay.fr/competitions/8293), and the evaluation library is available on GitHub (https://github.com/tonifuc3m/drugprot-evaluation-library). The DrugProt corpus, Large Scale corpus, Silver Standard Knowledge Graph and annotation guidelines are available on Zenodo (https://doi.org/10.5281/zenodo.4955410). Besides, the participant codes can be accessed through the BioCreative webpage (https://biocreative.bioinformatics.udel.edu/tasks/biocreative-vii/track-1/). The code to download the PubMed records for the large-scale corpus is available on GitHub (https://github.com/tonifuc3m/pubmed-parser), and the document selection criteria are available on Zenodo (https://doi.org/10.5281/zenodo.5656991).

## Supplementary Material

baad080_Supp
